# The Hospital. Nursing Mirror

**Published:** 1898-05-28

**Authors:** 


"The Hospital" Nursing Mirror.

Being the Nursing Section of "The Hospital."

[Contributions for this Section of "The Hospital" should be addressed to the Editor, The Hospital, 28 & 29, Southampton Street, Strand, London, W.C., and should have the word "Nursing" plainly written in left-hand top corner of the envelope.]

### News from the Nursing World.

### WESTMINSTER NURSING HOME AND WESTMINSTER HOSPITAL.

The vacancy caused by the resignation of Miss Pyne of the Matronship of these institutions has not yet been filled. The committee of the home has selected a lady who has previously held the office of matron at a county infirmary and a hospital under the Metropolitan Asylums Board. The House Committee of the Westminster Hospital, however, have not yet made any appointment, and we therefore do not mention the lady's name pending their decision in the matter.

### SOCIAL GATHERING AT THE NEW HOSPITAL FOR WOMEN.

It is customary to give a yearly "At Home" at the New Hospital for Women, Euston Road. This year the very pleasant gathering took place on the 24th inst., a little earlier in the season than usual. The hospital and wards looked charming, an abundance of flowers turning the place into a series of bowers. Tea was served in the doctor's library, and the visitors were escorted through the wards by members of the hospital staff. Mrs. Garrett Anderson, Mrs. Scharlieb, and others were present. Much interest was taken in the progress of the new nurses' home, the walls of which are rapidly rising. Considerable time, however, must still elapse before the new domicile is ready for occupation, when further structural improvements will be commenced.

### THE "LUCINA" BAZAAR, DUBLIN.

A bazaar in aid of the funds of the Rotunda Hospital in Dublin was held during last week. The nurses' stall realised from £17 to £20 for each of the five days. When all the money from raffles is called in it is probable that the total will be greatly augmented. Miss Ramsden, the lady superintendent, provided dinners, of which over 150 guests partook each evening. The dining-room for the occasion was the stately entrance hall of the hospital. The nurses took the part of waiting maids.

### AFFILIATED BENEFIT NURSING ASSOCIATIONS.

A conference of committee members of the Affiliated Benefit Nursing Associations, for the supply of cottage nurses on the Holt-Hockley system, was held, by permission of Lord and Lady Ancaster, at 12, Belgrave Square, S.W., on Wednesday, the 25th inst. The Earl of Gainsborough occupied the chair, and referred to the great growth during recent years in the number of Benefit Nursing Associations. When the central office of these associations was formed, four years ago, there were only 60 institutions in existence, now there were 114. Mrs. Garrett Anderson spoke in general approval of the lines on which the Benefit Nursing Associations were worked.

### COUNTY COUNCILS AND NURSE TRAINING.

Much interest is being displayed at the moment in the position of county councils in regard to the training of nurses for work in country districts. County councils have power to make grants for technical education, and several of them have already used this authority to give scholarships and grants to assist in the training of nurses in district work. Amongst these may be mentioned the Northumberland, Kesteven, Holland, Lindsey, East Suffolk, West Suffolk, Oxfordshire, Shropshire, East Sussex and Cambridgeshire County Councils. Some of these councils have hitherto paid the grants or scholarships direct to the nurse candidates, but Lady Ancaster and others strongly object to this course, which is followed by the Northumberland, East Suffolk, and some, if not all, of the Lincolnshire County Councils. Another plan is to pay the grants over to the association by which the nurse is trained. This latter course is adopted by the Shropshire, West Suffolk, and East Sussex County Councils. There is reason to believe that much more will be done in this direction by county councils in the near future.

### GUY'S HOSPITAL TRAINED NURSES' INSTITUTION.

In view of the increased accommodation which, it is hoped, will be built during the present year for the nurses of Guy's Institution, the managers have made arrangements with the hospital to enter twelve probationers yearly for training, instead of six as hitherto. So far, the number on the staff has varied between 85 and 90, but in the immediate future there will probably be many more. The number of applications for nurses was 1,208, that of the cases nursed 880—71 less than in 1896. The nurses have earned £6,763, they have received £3,421, and £621 has been paid into the Royal National Pension Fund on their behalf. Thirty nurses who were standing to participate in the bonus each received £3 10s. The bonuses are invested in the R.N.P.F., and will be available when the nurse reaches 55 years of age. One nurse, whose health failed some little time before attaining the stipulated age, has been pensioned by the managers at the rate of 10s. a week. Arrangements have been made with the Governors of the Bethlem Hospital for the nurses to receive training in mental cases; with the Metropolitan Asylums Board in infectious cases; and they are trained in midwifery by the lying-in charity of their own hospital. Two district maternity nurses are maintained, and the income for the Samaritan fund attached to this charity was £58.

### CATHEDRAL NURSE AND LOAN SOCIETY.

On the 18th inst. a popular and successful amateur dramatic entertainment was given at St. George's Parochial Hall, Newcastle, on behalf of the Cathedral Nurse and Loan Society. The pieces chosen were "The Nettle" and "The Coming Women." The acting was good, and there was a large and appreciative audience.

### CARLISLE DISTRICT NURSING ASSOCIATION.

The Executive Committee of the Carlisle District Nursing Association are much pleased at the formation last year of the Cumberland County Nursing Association, because of the impetus it gives to district work. The report of the Carlisle Society discloses an increase of work within its area; the nurses are very busy, and they work chiefly through and under the direction of the medical men. The only thing to regret seems to be the wasting of the balance in hand from £113 to £67. We note two excellent rules for the nurse. One is that her working day is regarded as eight hours long only, and that Sunday is to be a rest day as far as possible, only urgent cases claiming her attention then. Of course, it is impossible always to limit the hours of duty for a nurse, but, if eight hours be regarded as her normal day, provision would have to be made for any cases that constantly demanded more.

### PROBATIONARY NURSES.

All boards of guardians are not yet clear as to the signification of the Local Government Board definition of the term "a training school for nurses," nor the extent and limit of their own powers under it. It is their duty to inform themselves fully upon the point, as by acting in ignorance or carelessness they may inflict serious loss on those who serve them. Take, for instance, the proceedings of the Atcham Board when engaging two probationary nurses. It was evident that members of the Board knew neither the limit nor extent of their powers in the matter. They were somewhat doubtful of their ability to give certificates qualifying those who held them for the post of superintendent nurse, yet they could not exactly see why they should not. One member was troubled about the superintendent's capability to give the necessary instruction, another as to whether the medical officer would charge for his lectures, a third about the number of beds. Under the circumstances they did the only thing fair to the young women, namely, they told them that they engaged them on trial, that they could not say whether or no they could grant certificates, and that they might require them to bind themselves for three years at the end of three months. It is to be hoped that Mr. Dansey's suggestion to refer the scheme to the Local Government Board will be followed, and that before the expiration of the period named definite information on the subject will be afforded to the probationers.

### HOW TO TREAT THE NURSE.

Lady Margaret Vane, in "The Autocrat of the Sick Room" in the May number of the *National Review* rightly throws the blame of some failings of which nurses are accused upon the public. She very wisely says that people should use discrimination in choosing nurses, and not treat all as if they were on the same level. Nor, in her opinion, should the lady at the head of the household abdicate her position. For whilst she ought to treat the nurse with politeness and consideration, she ought not selfishly to neglect the patient because there is a nurse in attendance. If, says she, women did their duty towards their own sick menfolk, we should not hear so often of the patient making his nurse his bride upon his recovery.

### DISTRICT NURSING AT STOCKTON.

Five hundred and ninety-two cases were attended by the nurses of the Stockton and Thornaby District Nursing Association. Unfortunately, there has been much illness prevalent, but Miss Kidd and her staff have exhibited untiring energy in grappling with it. Their work in the homes of the poor has been supplemented by the purchase of tickets for the Ropner Convalescent home, which has enabled the association to send convalescents away for a change. Thanks to the efforts of a ladies' committee, new and commodious premises for the home have been secured in an admirable position. Owing to trade depression the workmen's contributions have fallen off, and there is a balance against the society of £6 8s. for the year. The income was £283. The total deficit is £62.

### TELEGRAPHIC ADDRESS.

It will be of interest to our readers and correspondents to know that the registered telegraphic address of the Scientific Press is "Ospedale, London." These two words will in future be all that will be required in addressing telegrams either to The Hospital or The Mirror or The Scientific Press from any part of the United Kingdom, and with the addition of the word England from any part of the world.

### SHORT ITEMS.

The committee of the Maryhill (Glasgow) Nursing Fund have decided to appoint a second permanent trained nurse. —The inspector of Queen Victoria's Jubilee Nurses' Institution reports thus of the Naas District Nursing Association: "It is a pleasure to inspect this district; everything is in such excellent order." The salary of the nurse (Miss Walshe) has been raised to £35 a year. The total income was £116, and there is a credit balance of 19 guineas —Hundreds of American women are offering their services as nurses for the war. But, so far, it has been decided that no women shall be permitted in the front.—The committee of the Bognor United Nurse Fund presented its second annual report on the 19th inst. The society has 10s. 10d. in hand, and 15s. more to come; it is, therefore, solvent and happy. Sixty-three sick persons were nursed during the year.—The three lady nurses for the Punjab Up-Country Nursing Association arrived at Kasauli last month.—Sutton-in-Ashfield Sick Nursing Association has 1,000 subscribers, and the balance in hand is £40. Nurse Robinson, who gives general satisfaction, has visited 208 cases during her second year's work.—The Thanet Guardians have recently paid Miss Coghlan £100 in settlement of her claim against them. Miss Coghlan was superintendent nurse, and became ill through an escape of gas.—The fourth municipal hospital at Berlin, of which the plans have already been drawn, will be ready for occupation in 1903. It will cost £650,000, will cover 105 acres of land, and will consist of 62 buildings, one of which is a training school for nurses.—The Duke and Duchess of Sutherland have lent Stafford House on June 9th for a grand concert in aid of the South London District Nursing Association. Several noted singers have already promised their services.—Mr. Robert Smith, J.P., C.C., has added £5 a year to the salary of Miss Ingham, in recognition of the meritorious way in which she has discharged the duties of Queen's nurse during the last two years at Longridge. —Miss Edith Helena Cordner, who received her training at St. Lucy's Hospital, Gloucester, passed the examination for inspector of nuisances held this month by the Sanitary Institute.

### Lectures to Surgical Nurses.

By H. A. Latimer, M.D. (Dunelm), M.R.C.S. of Eng., L.S.A. of London, Consulting Surgeon, Swansea Hospital; President of the Swansea Medical Society; Lecturer and Examiner of the St. John Ambulance Association, &c.

### XXXIII.—THE METHODS OF ANTISEPTIC AND ASEPTIC SURGERY, AND RESULTS EFFECTED BY THEM—THE OCCASIONAL ILL RESULTS.

In my last lecture I was speaking of antiseptic surgery. I am old enough to remember the introduction of this method, which we owe to the genius of Lord Lister. Before this great surgeon introduced his methods of operating and of dressing wounds we were constantly baffled in our work by septic processes which affected our patients, and hospitals had to be emptied from time to time on account of epidemics of erysipelas and pyæmia which prevailed there; and since we have learnt how to ward off these preventable diseases surgeons have grown bolder and bolder in their work, until regions which were formerly only operated upon most rarely, and then only by exceptional men, are now freely dealt with, when diseased, by every sort of surgeon who aspires to use the knife. The numbers of lives which have been saved through the work of Lord Lister in his introduction of this system of antiseptic surgery is incalculable; and if we add to these lives the numbers of people who have escaped losing limbs after injuries sustained by them, or when disease has affected them, we have a sum total of benefit accruing to mankind which, had his labour lain in the direction of destruction, as a general, with an equal result to his efforts, would have raised him to the highest dignity in the State and to the loftiest rank in the peerage. It is a comfort to feel that his fame will rest on higher claims than those of titles, and that whenever the history is written of the revolution of systems in surgery whereby the Victorian age has left an abiding splendour, the name of this great thinker and practical worker must stand out in imperishable renown. For go where we will over the civilised world we find this principle of warding off and destroying the germs of disease being practised, and although the very methods he at first adopted have been modified or altered, the idea is always the same, and the objects striven for are those which he inculcated. And these objects, as I have already told you, are the protection of the body against the hidden foes called bacteria, or microbes. It matters little whether by sterilising instruments and dressings before they are used we succeed in preventing the access of germs to wounds, or whether by the use of destructive agents we prevent them developing in injured parts, and so producing disease. The idea is the same. They and the poisons evolved by them are combated, and the good result follows. I will refrain from indulging in a retrospect of the various antiseptic methods which have been in vogue, as these lectures aim at being practical ones, and deal only with issues of present importance to you; so I will content myself with giving a description of what is done nowadays when the surgeon is about to operate, and what is done to rob septic wounds of their poisonous qualities, should such conditions prevail. To bring the matter home to you fully, I will ask you to figure to yourselves the removal of a breast for some tumour, an illustration which I have chosen for my purpose as this is an operation which is very frequently practised in the hospital. After the preparation which I have spoken of has been carried out, the patient is brought down to the operating theatre, and put under the influence of an anæsthetic. The surgeons who will be engaged in the case bare their arms, and wash these and their hands with great care, attending, in particular, to the condition of their nails to make sure of these being perfectly clean. The instruments which are to be used are taken out of boiling water, in which a small quantity of an alkali has been dissolved, and are placed in a metal dish in which an antiseptic lotion of 1 part of carbolic acid to 40 parts of water awaits them; and the sponges, which have been sterilised by a process of steaming performed beforehand, and which are kept after that preparation in a lotion of 1 in 20 of carbolic acid lotion, are taken out of the jar in which they have been placed, and are kept, ready for use, in a weaker solution of that antiseptic, from which they are wrung out when they are required by the surgeons. A towel wrung out of carbolic acid lotion is now placed near the operator, so that if he lays down any instrument at any time during his operation it may be on this purified receptacle; and the operation is commenced and proceeded with. During the operation the parts which are being dealt with are from time to time douched with an antiseptic lotion which is made to play upon them from the nozzle of a douche which contains a supply of that fluid, and if the operator's hands become soiled they are from time to time washed in some antiseptic fluid. Finally, one or more of the gauzes or cotton wools, or wood-wools, which have been impregnated with antiseptic substances, are applied to the wound, after all bleeding has been arrested, and its edges have been brought together with ligatures and sutures which are aseptic in character, and the operation is completed. By the use of sterilised instruments and sponges the aseptic part of the business has been carried out; by the employment of lotions of carbolic acid, or corrosive sublimate, antiseptics have come into play. Hereafter, with the majority of surgeons, these last are used when dressing the wounds, so as to destroy any micro-organisms which may have alighted on them; but some practitioners doubt their usefulness, and content themselves with the free use of water which has been sterilised by previous boiling. Moreover, it must be remembered that many surgeons after thoroughly cleansing the field of the operation only use sterilised water afterwards, and allow no antiseptics to come in contact with the wound. This is called aseptic surgery.

As to the antiseptics which are employed, their name is legion, there being a great many substances which are inimical to the life of germs. Carbolic acid, boracic acid, perchloride of mercury, permanganate of potash, solution of coal tar, and iodine, are those most frequently used, in solutions of various strengths; and iodoform and boracic acid are often applied in the dry state, as dusting powders. Carbolic acid and perchloride of mercury (corrosive sublimate) in appropriate strengths are undoubtedly very valuable germicides, but the latter has the disadvantage of staining steel instruments with which it comes into contact. As a dusting powder I have for some years past used a mixture of a small quantity of iodoform with a large quantity of boracic acid, and I am satisfied that this is a good preparation, and useful for the purpose to which it is applied. I have, however, known iodoform produce a marked amount of irritation when applied to wounds, which is never the case when the other substance is used.

You ought to know what evil results may occur to the system generally by the absorption of carbolic acid or of the mercurial preparation. In the case of the former substance it is apt—but very rarely—to set up vomiting and purging, faintness, and unconsciousness, symptoms of its absorption which are preceded by the patient's urine turning to a smoky brown, or even black colour; when mercury affects the system it does so by causing salivation, diarrhœa, and trembling movements in the limbs. But you will hardly ever meet with such instances of poisoning by these drugs, the cases being very few where they are used in sufficient strength to produce such untoward results. Should, however, any such symptoms declare themselves, you will know that they demand the immediate attention of the surgeon, so that a treatment may be adopted for curing the patient without delay. Fortunately, this cure is soon brought about by discontinuing their use, and by some simple measures being adopted for neutralising the effects wrought by them. As these measures do not lie within your province, I will not discuss them.

### Post-Graduate Clinics for Nurses.

By a Trained Nurse.

### ARTIFICIAL METHODS OF FEEDING.

### Rectal Feeding.—I.

The art of feeding is by no means comprised in the periodic administration by means of a feeding cup or tumbler of stated quantities of nourishment.

The nurse who prides herself on being trained must realise that the methods of artificial feeding are many, and that it by no means follows that because a patient is not able to take much, or any, food by mouth, that this necessarily involves a subsequent semi-starvation. The various methods of artificial feeding will be described and enlarged upon in the present series, so that the nurse may have at her finger ends the latest discoveries, the newest methods and the most approved way of nourishing her patient by artificial means, when the natural means for some reason fails to be capable of performing its office.

In the nursing of all cases it is most important that the nurse should keep watch and ward as to whether her patient is taking enough nourishment. In special cases attended with difficulty or pain in swallowing—in cases of persistent nausea, or when the patient through want of appetite, hysteria, or from disturbed mental conditions exhibits a distaste for food, it becomes doubly the duty of the nurse to inform the doctor. Delays are dangerous, but in such cases, when the loss of a few days' adequate nourishment may "run the patient down" below rallying point, it is fatal. In hemiplegia, for instance, or in throat paralysis after diphtheria, the patient may really be more than half starved unless his nurse knows enough of dietetics to realise that a few stray spoonsful of milk and beef-tea do not constitute a satisfactory form of nourishment. It is only the nurse who actually *knows* of the food which is rejected so soon as it is put into the mouth, and of the fact that a patient is too exhausted to swallow, or that he is unwilling to do so, because the act of deglutition is attended with a more or less degree of pain. The doctor noting the increasing exhaustion of his patient, the failing pulse, &c., may find out that sufficient nourishment is not being taken. But he should not need to make the discovery; by the time such symptoms of exhaustion actually show themselves so as to be diagnosed by the doctor, too much valuable time has been lost. The responsibility of reporting from the very first hour when the patient—be the reason what it may—ceases to take plentiful nourishment, rests with the nurse.

The doctor then proceeds to give instructions as to which method of artificial feeding is to be adopted, and the way of the nurse is clear. As most nurses have experienced, many patients, from sheer want of appetite, fall into the habit of taking far less than is needed to support life and restore convalescence, and such patients are badly off indeed if their nurses are not sufficiently instructed in dietetics to know just what constitutes under-feeding. There may not be so wide a difference between "enough" and not "quite enough," but the difference is of a kind to make it a very serious necessity that the nurse shall be able to judge between the two conditions.

There has grown up, somehow, a by far too strong belief among nurses that rectal feeding is more or less a last resort, that patients soon begin to reject nutrient enemata, that very little nourishment is absorbed by the bowel, and that this, therefore, is a rather hopeless method of sustaining a patient. I am specially anxious to point out that it lies very much with the nurse herself whether rectal feeding proves a success or no. There are very many points with regard to this method of feeding, and so much depends on their careful observance and delicate carrying out that I wish to make these papers most helpful and practical. At the same time I must emphasise the thought that no branch of sick dietetics can be learned absolutely by reading. The writer can put the student on the right path, and she can then study for herself by experience and by following the suggestions offered. But it is only those who are interested in food and sick cookery who will profit by the teaching. For one may prepare a pool of knowledge and conduct the students to the edge, but one cannot make them drink and assimilate of the water of learning, unless it pleases them so to do, and those who are not really fond of dietetics are certainly rather apt to fail in this most important branch of nursing.

I can assure every nurse that once the study is entered into *con amore*, dietary and sick cooking prove of the keenest interest. It is only the alphabet of the subject which is not alluring.

One advantage connected with rectal feeding lies in the fact that no idiosyncrasy on the part of the patient interferes with the administration of the food.

It is what he needs, not what he likes, that the nurse must consider. No question of appetite is involved, no personal inclinations count. Dietetics and science for once have full sway over the feeding of the patient, and regularity in giving nourishment can be employed without waiting till the patient is "inclined" to take it.

Nutrient enemata may be employed either to supplement the amount of food a patient is taking by mouth, as in gastric ulcer, peritonitis, enteritis, &c., or as an exclusive method of feeding. In cases of œsophageal cancer, or persistent vomiting, the patient must rely entirely on rectal feeding, as also in severe throat paralysis after diphtheria—though in the latter case throat feeding by means of a tube passed through the mouth into the œsophagus may be employed. In children suffering from diphtheria, or with bad scarlet fever throats, the amount of nourishment they consent to swallow may be, and often is, entirely insufficient for their needs. The quantity and quality must therefore be made up by rectal or nasal feeding.

Patients suffering from delirium tremens, the cataleptic, the hysterical, and the paralysed, must be included among the list of patients who may need rectal feeding.

The absorbing power of the rectum has its limits, but judiciously used and carefully given a patient may be sufficiently nourished by means of nutrient enemata to sustain life for from four to seven weeks, and even longer. Thus a serious crisis may be tided over; but, to enable a patient to gain the benefit of such prolonged rectal feeding, demands much skill and care from the nurse in charge of the case. The practical side of the question, and the means whereby an irritable bowel may be coaxed to retain and absorb the food thus administered, will be given in subsequent papers.

### Appointments.

### MATRONS.

The Isolation Hospital, Mogden, Middlesex.—Miss Annie Carter, ward sister of the Lewisham Infirmary, was appointed Matron of this hospital on the 20th inst. She received three years' training at the Devon and Exeter Hospital, and was afterwards sister at the Suffolk General Hospital.

### Minor Appointments.

Cancer Pavilion and Home Hospital, Hospital Gardens, Manchester.—Miss Annie Hamilton, of the Royal United Hospital, Bath, was elected Matron of this hospital on the 20th inst.

### Nursing in Paris Hospitals.

### B.—TRAINING LAY NURSES.

### IV.—Special Instruction.

Besides the two chief departments of training in the new schools—the theoretical and the practical—various sorts of other special instruction have to be given. Although the insane are, in France, departmental, and not municipal, charges, just as in England they are (or were until the Act of 1891, and still in the main are) county, and not parochial, yet the Department of the Seine manages to board a large number of the insane in La Salpêtrière and Bicêtre Refuge Asylums, and these are tended by nurses employed by the Assistance Publique. Thus, a section of the nurses have to be specially trained in asylum work. Again, the municipality offers special training to midwifery, although, strange to say, it gives no such special training to the nurses of its own lying-in hospital, La Maternité. However, the special training in midwifery I will deal with by itself in the two following articles. Another form of special instruction is of an elementary sort, required by the exigencies of the new situation.

The uncultured character of the great influx of lay nurses into the Paris hospitals since 1878 has necessitated some provision for making them more fit for positions of trust than before was necessary when the nursing sisters had all the responsibility. As adjuncts to all the four professional schools—La Salpêtrière, Bicêtre, La Pitié, and Lariboisière—it has been found necessary to establish primary schools to prepare the more uneducated nurses for entry into the regular course. In fact, in one instance the primary school was instituted before the professional school. The Lariboisière primary school preceded the professional by two years. The first primary schools, nevertheless, at La Salpêtrière and Bicêtre were thus located because a teaching staff already existed, coupled with the fact of the large number of nurses in each establishment.

These primary courses are, however, utilised to some degree, for professional training. Thus the 27 scholars in the primary school at Bicêtre last year were set to copying translations of Dr. E. J. Domville's "Manual," and also the "Manual" of the Medico-Physiological Association, instead of the ordinary tasks allotted to juvenile aspirants in orthography and caligraphy. A similar practice has been followed at the other schools from their inauguration.

Supplementary primary schools have also been instituted at Berck-sur-Mer, at the Foundling Hospital, and at the Tenon Hospital. Attendance not being obligatory, I cannot say that these are very successful. Thus at Tenon, a large hospital with a staff of 194 nurses, the primary course was started in October, 1896, with only 19 scholars (4 male and 15 female), which number had fallen to 10 at the annual examination in June, 1897, of whom only 7 were deemed worthy of primary certificates. This hardly indicates that the small proportion of scholars is accounted for by the superior education of the Tenon nurses. There was also an elementary course attended by 13 male and three female nurses. The hours for these courses were, in the primary one, three times a week in the autumn and spring, and daily from January 1st to April 1st, from eight to nine p.m.; and the elementary course three times a week, from half-past one to three p.m. for night nurses, and eight to half-past nine p.m. for day nurses.

One very curious reason for the institution of these primary schools was that many of the predominant class of Breton nurses in the early laic enlistments could not speak French, knowing only their native Keltic speech. As I have stated in the article on recruitment in Series A, this proportion of Bretons has, however, greatly lessened of recent years. In 1894, according to a partial census made by Dr. Bourneville in La Salpêtrière, of 170 female nurses 46 were born in Paris, 41 in Brittany, 9 in Savoy, 7 in the Vosges, 6 in the Seine et Marne, 5 in Cautal, 3 in Aveyron, and the rest in 30 other departments. At La Salpêtrière the primary school is much more attended than at Tenon, having over 100 scholars. Of course, it is a much larger establishment, but by no means in this proportion.

The most vexed question concerning special instruction is of that certain class of scholarships which the exigencies of the lay campaign induced the laicisers to establish in 1885, but which has now become a sort of Frankenstein to them. These scholarship holders, or boursières, were introduced because it was found that enough qualified matrons and assistants could not be found to take the places of the discharged sisters at the rate the vacancies were being made. This was especially the case as it was not found practicable to make the new or old lay nurses of lower grade obliged to attend the new schools and qualify themselves for promotion. The regular hours of the nurses are so long that the attendance out of work hours at school must be left a voluntary matter, though inducement to promotion may act as a powerful incentive. In the heat of the laicising campaign the Municipal Council was induced to appropriate 20,000 francs a year for 20 boursières to pursue the studies for nursing certificates without being obliged to perform special duties.

The primary schools are one of the great nuisances of laicisation. Owing to the inequality of instruction in the new lay nurses, they are inordinately expensive compared with the result obtained, such a great number of classes having to be made for a small number of scholars in each instance. Were it possible to refuse all recruits to the hospitals who could not pass a certain standard of education, all this would be avoided.

Another expense entailed by the lay nurses is brought about by the permission to have husbands and families. In all hospitals where children of nurses are found, special schools for infants have to be maintained. Of course, as France now maintains universal compulsory education, the children would have to be educated somewhere; but it is, of course, both inconvenient and expensive to have to maintain such exotic institutions as infant classes in the hospitals.

Finally, the last form of special instruction, one I have already mentioned in the first article of this Series B, is one not in *esse* but in *posse*. For several years back Dr. Bourneville has been hammering away at the matter of turning the school at La Pitié into a nurses' finishing school (*école de perfectionnement*), and as the Doctor has hitherto eventually succeeded in inducing the Municipal Council to accede to all his other plans of reform, he may, before long, have also his way in this, although, as I have said, the alarm and the increasing cost of laicisation may block the way this time.

Edmund R. Spearman.

### Central Bureau for the Employment of Women.

### MEETING AT WIMBORNE HOUSE.

On May 20th a largely-attended meeting was held at Wimborne House with the object of explaining the aims of the newly started "Central Bureau for the Employment of Women" (in connection with the National Union of Women Workers). The chair was taken by the Right Hon. James Bryce, M.P., and on the platform were Mrs. Creighton (who is the chairman of committee), Sir Robert Giffen, K.C.B., and Mrs. Bryant, D.Sc. Sir Walter Besant had promised to be present and to speak, but was, to the universal disappointment, at the last moment prevented from fulfilling his intention.

In spite of wretched weather Lady Wimborne's beautiful room—which is so often generously lent for the public service —was crowded, and amongst the audience might have been recognised many well-known "women workers."

In his opening speech the Chairman, after a passing allusion to the national loss in Mr. Gladstone's death, which was filling all thoughts, declared his sympathy with the scheme of the Central Bureau, and his conviction that the well-being of the nation depended on the well-being of its women. He explained that the object of the Bureau was not merely to establish one more amongst many agencies for finding work for women, but to bring together and strengthen those already existing. There was at present a great want of co-operation amongst the various agencies, and the idea of the Central Bureau was to centralise these isolated attempts to cope with a great difficulty, and to bring them into useful relation one with the other, by helping them to know what each was doing. The founders of the scheme had also been struck by the great lack of preparation and training amongst women who were compelled to earn their own living, and thought that the incompetency often complained of frequently arose because women sought work for which they were not fitted, not realising that it was better to do humble work well than attempt that for which they were not trained. There could be no more useful work than to find out how this training was to be obtained and be then able to advise those needing it how to set about its realisation. The Central Bureau would enforce the need of training, would endeavour to investigate employments, to advise employers as to the work for which women were properly fitted, and to be a medium of communication between employers and employed. Its work would not clash with that of the Industrial Council, which dealt with rather a different class of women workers, chiefly in the sphere of manual labour. It charged a moderate scale of fees, and hoped in time to become self-supporting; the names of those who were undertaking its management being a guarantee of economical administration.

Mrs. Creighton followed with a more detailed explanation of the scheme, which was, she said, due in the first instance to Sir Walter Besant, who, like so many others, had been greatly struck by the number of poor ladies forced in later life to work for themselves, and ignorant of the way to set about it. He had seen, too, how many women who tried to work with their brains had no one to stand up for their interests, how they were miserably paid, and powerless to help themselves. He hoped to see the scheme become a great national one, and he suggested to the National Union of Women Workers that they should take it up. They had begun in a small way, and early this year had ventured to take an office and make a start, looking forward to the day when the idea might culminate in a large and magnificent institution watching over the interests of working women. The Bureau hoped to be a great centre of information on all questions of women's employment, to be always ready to investigate new openings for women, to find posts for workers and workers for posts, not to overlap the work of other agencies, but when people came for information to know exactly where to send them for that information; not to sink into a registry, or to do work done by other agencies, and, finally, to protect the interests of women who stood alone and could not fight for themselves. It might be thought that when women took up new employment there was a danger lest men should be ousted from the field, but this was not really so. One of the new developments of modern days was the perpetual arising of new wants and needs. How many women now employed private secretaries, a want quite unknown to our grandmothers. Mrs. Creighton went on to speak of the number of young women who took up unsatisfactory work, of the hundreds who would answer an advertisement for a companion, while if a competent laundry matron was required she could not be found. Coming to the question of maintenance, Mrs. Creighton said that the work of investigation which the Bureau hoped to carry on would demand money; there were rent and salaries to be provided, and for help towards this they must ask for contributions from the public—£400 or £500 a year would be needed to do the work and keep up the office. It was proposed to start bureaux in large towns with which the Central Bureau would be in communication; and where a town was not able to support such a bureau, their endeavour would be to find ladies to act as correspondents, and so keep up the connection. Mrs. Creighton concluded by asking her audience to help the work of the Central Bureau by making use of it.

Sir Robert Giffen said the society was starting on a practical basis. It was not a charitable undertaking; it aimed at enabling young women to earn their own living in an adequate manner and at bringing them up to occupy good posts. It was worked by women for women, and was all the more likely to be successful. As to the fear lest women should supplant men in the sphere of work and take the bread out of their mouths, there was no fear of that, though, if there were, he thought they had a right to do it. In his opinion, the increased competition resulting from the enlarged area of women's work was a good thing, adding to the productive power of the country.

The last speaker was Mrs. Bryant, who pointed out in a practical and convincing way the many hitherto unheeded methods in which girls of education might well be employed. Why, she asked, should not girls with artistic capacities become excellent heads of departments in shops, or those with a turn for domestic matters take up such work as housekeeping in flats, or managers in hotels and restaurants? Then there were also rich girls. Why should they not work also, and, by putting capital into the wealth-producing trades, do excellent work in the superintending of shops and factories, opening up more employment for women by-and-by? The rising generation would owe much to the Central Bureau for the efforts it would make to open up employments and disseminate information concerning them.

A very cordial vote of thanks to Lady Wimborne for the use of her house brought the meeting to a close.

All particulars concerning the work of the Central Bureau can be obtained from the Hon. Secretary (Miss Margaret Bateson), at the offices, 60, Chancery Lane, W.C.

### INCREASE OF RATIONS.

The Armagh Board of Guardians have ordered an increase of rations to their nurses. The diet scale for the week now stands as follows: Bacon, 3 lb.; eggs, 6; milk, 3½ quarts; butter, 1 lb.; bread, 7 lb.; oatmeal, 4 lbs.; potatos, 14 lb.; meat, 3½ lb.; 4 oz. tea; and 1 lb. sugar.

### Parents' National Educational Union.

II.—THE DIRECTION OF THE WILL AND DESIRES.

THE members of the P.N.E.U. met one afternoon during the recent Conference at 15, Belgrave Square, by permission of the Duchess of Bedford, when the chair was taken by Canon BARNETT, and Miss Mason gave an interesting address upon "The Direction of the Will and Regulation of the Desires."

Every human being, Miss Mason said, was endowed with certain natural desires, such as the desire for knowledge, the wish "to know," a sense divinely appointed for the encouragement of the intellect. Some people "wanted to know" in a different sense; they wanted to know what Mrs. So-and-So had bought lately, when the next millinery sale would take place, if they had ceased to want to know other and more important things. Then the whole desire had been misused, and the human being brought down to the level of that which it wanted to know. Therefore, should all gossip about people be avoided in the hearing of children, and in the nursery, and a wholesome curiosity about the "latest thing out" in the world of nature be stimulated and encouraged. Every child had a desire for society, a natural human desire, which might work for virtue or vice as it was directed. Children might be taught to select their friends, not those belonging to the "best connected families," but those with the nicest minds. Then there was the desire for approbation, a feeling harmfully played upon when nurses encouraged children to do such and such a thing because of what "so and so would think," or even that "God will not love you if you do it," a terrible idea against which she implored parents to be on their guard. This love of esteem was responsible for much loss of individuality. People furnished their houses, and even dressed themselves, not in expression of their own personality, but because it was the fashion, because of the way "things were done." Yet there was a certain wisdom in telling children to mind their elders' opinions—it taught them reverence; but this must not grow into too great a regard for public opinion. They must be given principles to live up to for themselves.

Everyone, too, possessed the desire of excelling; the wish to have everything the best of its kind, not in order to have the best thing in itself, but that no one else should have it better. There was a danger that this desire might ruin the intellectual power of the nation. And in connection with school life, it was to be feared that the questions asked by parents of the children concerning their work were not as to the books read and the things learnt, but "What marks have you got?" and "How have you risen in your form?" It was the fetish of the examination set up instead of the love of knowledge for its own sake. Boys and girls, she feared, did not now return home from school full of interest in the subjects they had been studying, not filled with a "divine curiosity," but eager to get the highest marks and take the highest place. Some children's individuality was strong enough to survive in spite of this iniquitous system, but in others it was quenched, and the desire for knowledge was suppressed and that of emulation took its place. It was no doubt want of thought rather than want of heart on the part of parents who permitted this state of things. The desire to excel might be directed to channels where it could only do good, the wish to be the most punctual, the most helpful, &c. ambition so directed helping to form a perfectly well balanced character. The desire for power, too, might be so regulated that it should work for good; let ambition be trained to be the ambition for "service," and so would the best men and women be sent out into the world.

Miss Mason went on to speak of the regulation of the desire for wealth; let children know from early years that money was not everything, was not the strongest force in the world; character was infinitely stronger. And then came the consideration of the force which must regulate all these desires; the will, backed by the trained conscience and inspiring spirit. And by way of a practical suggestion Miss Mason spoke of the wisdom of "changing the thoughts" of a child, to use an old nursery expression, from the thing which they ought not to do to the thing which they ought to do, and towards this end strongly recommended the cultivation of "hobbies" and some special recreation for every child.

### Workhouse Infirmary Nursing Association.

NURSES' ANNUAL GATHERING.

THE annual gathering of the Mary Adelaide Nurses was held on the 19th inst., by kind permission of the Hon. Mrs. R. Talbot at her house in Lower Grosvenor Street. It was unfortunately a miserably wet night, which accounted for the absence of a good many of the invited guests, but those who braved the weather spent a pleasant evening. The medals were to have been presented to the nurses who had earned the right to that coveted distinction by Mrs. J. G. Talbot, who was, however, unable to be present, in consequence of the death of Mr. Gladstone, her uncle. In her absence Lady Wantage gave the medals to the nurses. Miss Gill was there, and Dr. Savill, Dr. Downs, Miss Catherine J. Wood, Miss Amy Hughes, Miss Fynes Clinton, Miss Chapman, and Miss Wesley, of St. George's-in-the-East.

Dr. Savill opened the evening's programme with a short speech. He spoke of the great loss the association had suffered by the death of the Duchess of Teck, whose keen personal interest in the nurses who bore her name had continued to the last. Commenting upon the resignation of Miss Wilson on account of ill-health, Dr. Savill referred to her admirable and untiring work for the association during past years, and to the deep regret felt by all that she had been obliged to give up her post of honorary secretary. It was earnestly hoped that she would benefit by her stay abroad, and return once more able to take an active part in its interests. In conclusion, he wished to make it quite clear to everyone that the association was as fully alive as ever, and its nurses still in exactly the same position as before the meeting last December, at which it was decided to curtail the work of the Association as to the further training of nurses. The connection with those nurses who were attached to the association was maintained as fully as before, and would continue to be so.

An admirable address was afterwards given to the nurses by Miss Hughes on various practical points connected with poor-law nursing. She touched on the ever-recurring difficulty of the relation between the trained nurse and the untrained master and matron, and urged upon nurses that it was of no use endeavouring to reform them; the only possible plan at present was to accept the inevitable and make the best of matters as they were in spite of all discouragements and difficulties.

Some very enjoyable music followed, and at ten o'clock the nurses said good night to their hostess, and went off, armed as usual with the delightful country "posies" always sent for them on these occasions by Lady Wantage.

### Wants and Workers.

[THE attention of correspondents is directed to the fact that "Helps in Sickness and to Health" (Scientific Press, 28 & 29, Southampton Street, Strand, London, W.C.) will enable them promptly to find the most suitable accommodation for difficult or special cases.]

MISS BAKER, The Home Farm, Ranston, Blandford, offers a room at a nominal rent to tired-out infirmary or district nurses requiring change. References required.

"SISTER C.," The Cottage Hospital, Cumnock, N.B., is anxious to obtain a bath chair for the use of an invalid.

### Hygiene Made Popular.

It is of the utmost importance that hygienic truths should be brought home to the rustic mind, and that young girls should be taught the value of open windows and the dangers lurking in the dust-bin and the contaminated well. But, however careful such teaching, there must always be a "submerged tenth," to whom the gospel of health will be preached in vain. A few selections are given below from the written papers of some country girls competing for a scholarship in domestic economy.

The purpose of water filtration is thus lucidly explained by one girl, who states that "People, when the hot weather comes, filter their water so as to catch any infirmery in it." While another girl gravely points out that "dangers in water may be brought on by drinking dirty water and what has got little insects in it, which gives tiford fever." Another candidate grows quite prophetic at to the condition of the dust-bin. Probably she means something, but the diagnosis of her meaning is not clear. "No rubbish should be thrown in; I do not mean any all but decayed greens should, if it could be prevented, be thrown in. If people will not take notice of this advice they will have to suffer from the consequences of having fever; typhoid and all sorts of others are risen from the cause of bad drains."

As to ventilation the statement occurs that "the principal thing by which we get air into our lungs is by the window. Some people smuggle up their windows with pads so as to keep the pure air they made the excuse that it is bad for their lungs another they make out that it is bad for their rheumatics.

"Heat producers" are given as "hot broth, hot milk pudding, and potatos."

It would appear from the following that the teaching as to drains must have been somewhat mixed. "The best way to keep a drains which connects the house clean is to have nothing go down the pipe which connects the drain excepting water or any liquid, or else the piece of food, or whatever it is, would rot and throw out a bad smell, which would cause fever or any infectious disease."

And the philosophy of keeping food pure and free from contamination is distinctly a curiosity. "Many people when they go shopping buy things in little fiddling piece, enough to last for a day, but it is best to buy it in large quantities; it does not go stale, but keeps it in a constant moisture."

### Royal British Nurses' Association.

One hundred and twenty-two members attended the special general meeting of the Royal British Nurses' Association, which was held in the rooms of the Royal Medical and Chirurgical Society, Hanover Square, W., on Tuesday, 24th inst., to consider the alterations in the new bye-laws suggested by the Privy Council. Her Royal Highness the Princess Christian was present, and at her request Sir James Crichton Browne took the chair.

Mr. E. A. Fardon (hon. medical secretary) moved the following resolution:—

"That the bye laws appended hereto, which have been drawn to meet the objections made by the Privy Council to the bye-laws passed at the general meeting held on December 17th, 1897, be approved, and that the same be submitted to the Privy Council for their approval, and, if approved by the Privy Council, become and be the bye-laws for the time being of the Association; and that, if the bye-laws passed by the meeting are approved by the Privy Council, then as from the date of such approval the existing bye-laws be rescinded."

He said that he did not intend that afternoon to dwell upon the nature of the opposition which they had had to encounter in the passage of these bye-laws, nor upon the difficulties and delays which had arisen owing to that opposition. He would rather congratulate the Association on the fact that, in spite of the nature of that opposition, the Association had practically obtained the sanction of the Privy Council to the bye-laws as passed at the meeting in December last, and that the alterations suggested were very immaterial, and did not affect the principles upon which they were drawn up. (Applause.) There were three main points to which the Privy Council had drawn the Association's attention. First, the Council had pointed out that the Association was unable to appoint patrons or vice-presidents except as honorary officers. That was the intention of the new bye-laws, and so they had been altered to meet that point. Then the Council said that the bye-laws must fix the subscriptions of the members, and that any alteration in the amount in the future must be made by means of a new bye-law. To that there was no objection. Finally, the bye-laws laid down that the Corporation should be entitled to keep a register of persons qualified to act as nurses. The Privy Council objected to the term "register," as it might lead to the idea that nurses who were placed on the register held, in some way, a kind of State qualification. They suggested that the word "list" should be used, and that had been done in the bye-laws submitted to the meeting. There were a few other minor alterations, which the committee had accepted. In conclusion, he appealed to all the members of the Association—medical men, matrons, and nurses—to help the committee to get these bye-laws passed as soon as possible so that the Association could put behind it all that time of contention and difficulty through which they had passed, and really set themselves to do more earnest work for nurses than they had hitherto been able to do. (Applause.)

Mrs. Coster seconded.

Dr. Bedford Fenwick attempted to enter on a general discussion of the bye-laws, but was called to order by the Chairman. As Dr. Bedford Fenwick continued to speak, the Chairman put it to the meeting whether any discussion should be allowed except on the alterations suggested by the Privy Council. The meeting unanimously decided it should not, and eventually Dr. Bedford Fenwick sat down.

The motion was put, and carried with one dissentient (Dr. Bedford Fenwick).

The meeting then terminated.

### Novelties for Nurses.

### CLARKE'S "CRICKLITE" LAMP.

The question how best to provide light for an invalid is one which does not always obtain the consideration which it deserves. If, as we all know is the case, a bare and naked light is an aggravation and annoyance to patients whose nerves are on the strain, we may well believe, what in fact experience teaches us, that a shielded light which in its form and appearance is pretty and attractive may be not without its soothing influence in the sick room. For this reason, if for no other, the cricklite lamps, which are made by the same firm as supplies the well-known pyramid night lights, are worthy of consideration by those who have to do with the arrangement of sick-room furniture. There can be no doubt that they are peculiarly fitted for decorative purposes, and the delicate invalid will be all the better for feeling that she and her room are the centre of much that is "nice," instead of being oppressed by the thought, which to some is very real, that illness is repulsive, and that in this repulsiveness she has her share. For other reasons, however, these lights are to be recommended for the sick-room. A naked candle is irritating to the eye, while many patients are nervously fidgetty about lamps, and an ordinary candle with a shade is a positive danger about which fidgetiness is quite excusable. Now a cricklite light is a quiet, steady-going thing, regarding which no patient is likely to be nervous, even when left alone with it.

### Everybody's Opinion.

[Correspondence on all subjects is invited, but we cannot in any way be responsible for the opinions expressed by our correspondents. No communication can be entertained if the name and address of the correspondent is not given, as a guarantee of good faith but not necessarily for publication, or unless one side of the paper only is written on.]

### "THE HOSPITAL" CONVALESCENT FUND.

"L. H." writes: The postal orders reached me last evening. I am very thankful to you for the very kind manner in which you have helped me, and hope, when I am able to do so, to show my gratitude by contributing to the fund.

### THE COAST HOSPITAL, SYDNEY.

"Nurse M. P." writes: Pardon the liberty I am taking, but in The Hospital for the 21st I saw something about the Coast Hospital, Little Bay, near Sydney. I only returned last week from Australia, where I have been engaged in district nursing for nearly nine years. Most of my work was Government work, and many of my patients were sent to the Coast Hospital. Although I do not know the matron personally I do know the doctor. He is a thoroughly just man, one who would not allow the nurses to be overworked or to have too long hours. No one would say they were overworked who saw them coming into theatres and other places of amusement on the ambulance, for cabs are expensive. They are fat, healthy-looking girls. Little Bay Hospital is beautifully situated just opposite the bay. The wards and nurses' rooms open out into large airy verandahs. No one who knew it would speak of the nurses as being either overworked or uncomfortable. I have often seen half-a-dozen of them in a row at the theatre enjoying themselves. Indeed many nurses would gladly give their little fingers to have their posts. If you could do something to prevent English private nurses going out there it would do some good. Many private nurses are starving after having been out a few weeks. I was lucky enough to obtain employment in the home, but I had to work very hard and to walk long distances. The cases are often very bad, as there are no incurable hospitals. I have frequently nursed cases in wooden houses that were once stables. There are no parish doctors, so the district nurse has to be almost a doctor as well. Many times medical men generously went out of their way to give me help, the chemists would make up their prescriptions free, and the Government afforded charitable relief.

### POOLE UNION WORKHOUSE.

Miss J. C. Spence writes: I think it is only fair to the Day Nurse and myself to contradict the following statements made against us, the Superintendent and the Day Nurse, by the Guardians of the Poole Union Workhouse. The clerk read a report from the medical officer of the workhouse, who said that he had to complain that his instructions were not carried out with reference to a water-bed which was occupied by one of the female patients; that the superintendent nurse had also neglected to carry out his instructions with reference to the feeding of certain inmates. The clerk further stated that he had received a letter from one of the lady visitors, who stated that one of her colleagues on visiting the house had received universal complaints about the day nurse; she feared that some of the old and infirm women were not dealt with as they should be. One guardian could bear out the statement of the medical officer as to the state in which the woman was left, the one on the water-bed. I wish the guardians and the public to know that the patients, in so far as our power lay, had every care and attention, though the above would lead one to believe they were sadly neglected. The woman mentioned was always left in a clean dry condition, with a hot water bottle filled with boiling water twice a day, and as to the water-bed, each day as soon as hot water could be obtained, it was put into the bed, and I can truthfully say that not once was the water cold when it was drawn off. Then, again, as to the feeding of the patients, it had only been suggested the day of the committee meeting that the nurse should feed the helpless cases, but this had been done before any such suggestion had been made. Let me add that I did not send in my resignation because I thought I should have been asked to resign, for I have done nothing to merit such a request, and though the guardians may have the power to request, it is certainly beyond their province to "dismiss" any superintendent nurse. Why did they not publish what were the "other inconveniences" mentioned in my letter of resignation? One great inconvenience is, there is only one sink in which to wash every utensil that may be used in the sick wards, as well as all drinking pots, milk and tea cans, &c. Another great drawback is the lying-in ward, there being only one ward for labour and accouchement, and during the time I was there there were two confinements, one that of a syphilitic woman. Was this fair to the other woman and child? All dirty lotions, &c., had to be taken down stairs to the one lavatory which was used by all the other female patients, averaging twenty in number. Would medical men consider this safe? When I was asked my candid opinion of the place I mentioned some of the inconveniences, and one of the guardians asked "Did I expect to find an operating-room?" When one is met with such questions one naturally concludes there is no alteration thought of, and consequently I deemed it best to resign early.

### Where to Go.

Nurses' Missionary Society.—H.R.H. the Princess Christian has graciously consented to open a loan exhibition and sale of work for the above society at the house of the Society for Promoting Christian Knowledge, Northumberland Avenue, on Thursday, July 7th, 1898, at twelve o'clock.

Messrs. Cassell and Company's annual exhibition of Drawings in Black and White will be opened on the 25th inst. at the Cutlers' Hall, Warwick Lane, E.C., and contain pictures by J. MacWhirter, R.A., Alfred East, J. Pennell, H. G. Glindoni, H. M. and W. Paget, J. Charlton, Gordon Browne, H. A. Harper, W. H. Margetson, Sir J. D. Linton, T. Fulleylove, W. Hatherell, Hal Hurst, P. Tarrant, C. Burton Barber, W. H. Overend, and many others.

Medical, Surgical, and Hygienic Exhibitors' Association's Exhibition.—We would draw the attention of our readers to this exhibition, which is to be held from May 31st to June 3rd (inclusive) at the Queen's Hall, Langham Place, London. Everything of interest to the medical profession, and among them many things which nurses would like to see, will be well represented by the foremost manufacturing firms. An excellent programme of music has been arranged for every afternoon and evening. Nurses in uniform will be admitted. The stall of the Scientific Press is No. 98.

### Presentations.

Miss Z. Stevenson on leaving the Birmingham General Hospital, of which she had been matron for three and a-half years, received a large number of handsome gifts in appreciation of her work there, and as expressions of congratulations and good wishes on her approaching marriage with the Rev. Stanley G. Collier, M.A., amongst which were an illuminated address, a handsome silver tea service, and a list of subscribers from the committee of management; a silver salver and crumb scoop from the residents of the hospital, a gold bracelet from the nursing staff, and silver serviette rings from the servants and porters.

Nurse Hughes, who for some time has been engaged at the Middlesbrough Sanatorium, has been presented with a gold brooch and a silver medal by Superintendent Knowlson, on behalf of the members of the police force of that town. The medal bears a suitable inscription. The gift is in recognition of the great care rendered to Police-constable Bailey whilst suffering from a severe attack of small-pox.

### For Reading to the Sick.

### WHITSUNTIDE.

"If we live in the Spirit, let us also walk in the Spirit."—*Galatians* v. 25.

**Verses.**

It fills the Church of God; it fills
The sinful world around;
Only in stubborn hearts and wills
No place for it is found.

Come, Lord, come, Wisdom, Love, and Power,
Open our ears to hear;
Let us not miss the accepted hour;
Save, Lord, by love or fear. —*Keble.*

From Thy dwelling-place above,
From Thy Father's throne of love,
With Thy look of mercy bless
Those without Thee comfortless.

Now in glory Thou dost reign,
Won by all Thy toil and pain;
Thence the promised Spirit send,
While our prayers to Thee ascend.
—*Lati Hymn.*

When as the apostles knelt
At the third hour in prayer,
A sudden rushing sound proclaimed
That God Himself was there.

Forthwith, a tongue of fire
Is seen on every brow,
Each heart receives the Father's light,
The Word's enkindling glow.

The Holy Ghost on all
Is mightily outpoured,
Who straight in divers' tongues declared
The wonders of the Lord.
—*Hymns Ancient and Modern.*

**Reading.**

Fight the good fight of faith, lay hold on eternal life, whereunto thou art so called and hast professed a good profession before many witnesses.—1 *Timothy* vi. 12.

The Comforter, Which is the Holy Spirit, Whom the Father will send in My Name.—*St. John* xiv. 26.

Christ's prayer was, "Father, give them the Holy Spirit to teach, sanctify, and comfort them." His Father should send, He said; and His Father did send, and the Holy Ghost came, to-day. And came in that sort whereof they had most need—a "Comforter." If we ask, why under that term, to show the peculiar end to which He came. If they had been perplexed, "The Spirit of Truth." If in pollution of sin, "the sanctifying Spirit." But to day they were as orphans, cast down and comfortless, their hearts full of heaviness. It was comfort they wanted; a Comforter to comfort them all. —*Bishop Andrewes*

It was one special end why the Sacrament itself was ordained—our comfort. The Church so telleth us, "He hath ordained these mysteries, to our great and endless comfort." "The Father will give you the Comforter." Why He giveth Him we will see; how He giveth Him we know not. The means for which He giveth Him is Christ, His entreaty by His Word in prayer, by His Flesh and Blood in sacrifice, for His Blood speaks, not His Voice only. These the means for which; and the very same the means by which He giveth the Comforter; by Christ the Word, and by Christ's Body and Blood both. In tongues it came, but the tongue is not the instrument of speech only, but of taste, we all know. That not only by the letter we read and the word we hear, but by the Flesh we eat and the Blood we drink at His table we may be made partakers of His Spirit and the comfort of it.—*Bishop Andrewes.*

### Notes and Queries.

**The contents of the Editor's Letter-box have now reached such unwieldy proportions that it has become necessary to establish a hard and fast rule regarding Answers to Correspondents. In future, all questions requiring replies will continue to be answered in this column without any fee. If an answer is required by letter, a fee of half-a-crown must be enclosed with the note containing the enquiry. We are always pleased to help our numerous correspondents to the fullest extent, and we can trust them to sympathise in the overwhelming amount of writing which makes the new rules a necessity. Every communication must be accompanied by the writer's name and address, otherwise it will receive no attention.**

*A Town in Need.*

(79) Can you kindly tell me of a town in need of a good nursing home, (2) and what capital is required to start one on an economical scale?—*A Doctor's Daughter.*

It is impossible for us to help you in this. Experienced nurses so often fail in enterprises of the kind that we could not recommend anyone to undertake them. 2. About £1,000.

*Massage and Electric Treatment.*

(80) Is there a hospital or institution in London where massage and electric treatment can be obtained in return for services in wards in the same way as midwi ery is taught?—*Smith.*

We know of no institution that teaches either massage or midwifery on reciprocal terms.

*Infants' Diet Chart.*

(81) *Vincit Veritas* would like to know if there is an infants' diet chart published giving the number of feeds in 24 hours and the quantity according to age?

In the new edition just published of Chevasse's "Advice to a Mother" (J. and A. Churchill), which has been practically rewritten by Dr. George Carpenter, the following table is given regarding the feeding of infants at different ages:—

| Age. | Intervals of Feeding. | Number of Feedings in 24 Hours. | Average Amount of each Feeding. | Average Amount in 24 Hours. |
|---|---|---|---|---|
| First Week ... | 2 hours ... | 10 ... | 1 oz. ... | 10 oz. |
| First Month | 2½ ,, ... | 8 ... | 2 to 3 oz. ... | 16 to 24 oz. |
| Second Month ... | 2½ ,, ... | 8 ... | 3 to 4 oz. ... | 20 to 30 oz. |
| Third and Fourth Months ... | 3 ,, ... | 7 ... | 4 to 5 oz. ... | 30 to 35 oz. |
| Fifth and Sixth Months .. | 3 ,, ... | 6 ... | 6 to 7 oz. ... | 35 to 40 oz. |

*District Nursing by Midwives.*

(82) I am a country district nurse; my work is chiefly midwifery. Ought I to attend a case of cancer of the breast—advanced, and which has never been operated upon? Or am I risking my other patients by so doing?—*Sister Eva.*

The question is an important one. A surgeon who merely has to examine the case would probably not hesitate. If it were foul and suppurating he would touch no part until it had been cleansed with antiseptics. The probabilities are that he would not find it necessary to touch it at all except by an instrument, and by the free use of antiseptics afterwards he would be able to make himself safe again. It is very different with the nurse. The dressing of the wound is probably the least risky part of the performance, for by the free use of antiseptics and by using swabs and forceps instead of sponges it is sometimes possible to avoid direct infection of the hands. But neglected cases of cancer of the breast which are discharging freely are apt, both they, their clothing, and their bed clothing, to become saturated with pus producing organisms and septic dust, and it is extremely difficult for anyone who has to perform the duties of a nurse to avoid becoming charged with the general infection. We think Sister Eva had better get some one else to look after this case. We may add that the Local Government Board wil not permit the guardians to employ a district nurse who is at the same time doing midwifery.

*The Age of Probationers.*

(83) Will you tell me if the standard of ages at which candidates may enter the hospitals for training as nurses is unalterable? I am 19½ years old, and very strong.—*Anxious.*

You can only enter a children's hospital at that age. Why not study cooking, housekeeping, and anatomy until you are old enough to train?

*Nursing in Cairo.*

(84) Am I right in thinking that there is a private nursing institution in Cairo? I should be very glad of any information.—*Ramlek.*

Possibly the matron, Kasr el-Aini Hospital, Cairo, could tell you. Can any of our readers give Ramlek any help?

*The Nauheim Treatment.*

(85) Can you inform me how to be instructed in the Nauheim treatment for heart disease?—*Matron.*

The best plan is, of course, to go to the birthplace of the treatment—Nauheim—and study it there. You can also read such books as "The Schott Treatment of Chronic Diseases of the Heart," by Dr. Bezly Thorne, and practical instruction might be obtained at some of our English baths where this treatment is in vogue, amongst which may be mentioned Harrogate and Sidmouth.

*Nurses' Holiday Home.*

(86) Is there any prospect of a nurse with ten years' experience succeeding in establishing a nurses' home of rest at a seaside place? Could it be done for £300?—*M. C. J.*

There is little or no chance of success. The difficulties to be overcome are enormous, the capital too small, and the sort of nurses who, when on a holiday, are able to pay well, are likely to keep away from others of the same calling.

